# Predicting enzymatic function of protein sequences with attention

**DOI:** 10.1093/bioinformatics/btad620

**Published:** 2023-10-24

**Authors:** Nicolas Buton, François Coste, Yann Le Cunff

**Affiliations:** Univ Rennes, Inria, CNRS, IRISA—UMR 6074, Rennes 35000, France; Univ Rennes, Inria, CNRS, IRISA—UMR 6074, Rennes 35000, France; Univ Rennes, Inria, CNRS, IRISA—UMR 6074, Rennes 35000, France

## Abstract

**Motivation:**

There is a growing number of available protein sequences, but only a limited amount has been manually annotated. For example, only 0.25% of all entries of UniProtKB are reviewed by human annotators. Further developing automatic tools to infer protein function from sequence alone can alleviate part of this gap. In this article, we investigate the potential of Transformer deep neural networks on a specific case of functional sequence annotation: the prediction of enzymatic classes.

**Results:**

We show that our EnzBert transformer models, trained to predict Enzyme Commission (EC) numbers by specialization of a protein language model, outperforms state-of-the-art tools for monofunctional enzyme class prediction based on sequences only. Accuracy is improved from 84% to 95% on the prediction of EC numbers at level two on the EC40 benchmark. To evaluate the prediction quality at level four, the most detailed level of EC numbers, we built two new time-based benchmarks for comparison with state-of-the-art methods ECPred and DeepEC: the macro-F1 score is respectively improved from 41% to 54% and from 20% to 26%. Finally, we also show that using a simple combination of attention maps is on par with, or better than, other classical interpretability methods on the EC prediction task. More specifically, important residues identified by attention maps tend to correspond to known catalytic sites. Quantitatively, we report a max F-Gain score of 96.05%, while classical interpretability methods reach 91.44% at best.

**Availability and implementation:**

Source code and datasets are respectively available at https://gitlab.inria.fr/nbuton/tfpc and https://doi.org/10.5281/zenodo.7253910

## 1 Introduction

The number of protein sequences available in bioinformatics databases is growing at a rapid pace. For instance, the reference protein knowledge base UniProtKB (The UniProt Consortium 2021) grew from 180 million available sequences to over 225 million between February 2020 and February 2022. One of the major questions of interest is to bridge the gap between these sequences and the functions of the corresponding proteins (Schnoes *et al.* 2013). However, functional annotation supported by experimental data is a difficult, expensive, and time-consuming task (Zhou *et al.* 2019). Hence, automatic functional annotation of protein sequences has been a growing field over the past years mainly driven by sequence alignment-based methods (Baldazzi *et al.* 2021) and, more recently, machine learning approaches (Zhou *et al.* 2019).

The functional prediction of enzymes, which account for around half of all known proteins (The UniProt Consortium 2021), is an important application of functional annotation of protein sequences. This task is a testbed for the development of predictive methods for several reasons. First, the enzyme’s functions are well-defined according to experimental evidence. These functions are then standardized by Enzyme Commission (EC) numbers which provide well-defined targets to train machine learning models (Webb 1992). Secondly, the amount of annotated enzymatic sequences available is sufficient to enable training and independent large-scale evaluations.

This has paved the way for enzyme sequence classification by machine learning approaches (Borro *et al.* 2006, Qiu *et al.* 2009, De Ferrari *et al.* 2012, Matsuta *et al.* 2013, Volpato *et al.* 2013, Nagao *et al.* 2014, Dalkiran *et al.* 2018, Li *et al.* 2018, Strodthoff *et al.* 2020, Nallapareddy and Dwivedula 2021, Yu *et al.* 2023, Sanderson *et al.* 2023). In particular, Strodthoff *et al.* (2020) developed one of the state-of-the-art prediction tools based on sequences only. They used their deep learning model to automatically infer the relevant internal vectorized representations of the sequences (the ‘sequence embedding’), from which the EC prediction is derived.

In terms of architecture, they chose to base their language model on an ASGD weight-dropped long short-term memory (AWD-LSTM) neural network architecture (Merity *et al.* 2017). Architectures based on LSTM can handle long sequences by partially solving the vanishing gradient problem. Yet, they still seem to struggle to properly account for long-distance interactions which are known to be relevant in protein structures. In natural language processing (NLP) applications, such LSTM architectures are being superseded by a non-recurrent architecture: the Transformers. Transformers primarily use the ‘attention’ mechanism (Vaswani *et al.* 2017). Transformers seem to better account for long-range interactions as witnessed for instance by the success of BERT (Devlin *et al.* 2019) or T5 (Raffel *et al.* 2020) on NLP benchmarks.

In this work, we propose to evaluate the performance of Transformers for protein functional annotation based on sequences. The potential of Transformers applied to protein sequences has already been identified by several seminal studies: in their early paper calling for the development and proper assessment of better protein modeling methods, Rao *et al.* (2019) introduced five tasks. On this benchmark (TAPE), they compared classical methods using alignment-based features (Netsurfp2.0, RaptorX, and DeepSF for secondary structure, contact prediction, and remote homology, respectively) and multiple deep learning architectures, namely convolutional neural networks, LSTM, and Transformers. While Transformers outperformed the other methods on several tasks (fluorescence and stability), they were still lacking on others: homology prediction (LSTM performing better) and structure prediction (methods based on alignment-based features performing better). More recently, Elnaggar *et al.* (2022) have conducted a high-performance computational study of Transformers on large protein sequence databases to obtain on par, or state-of-the-art results on secondary structure prediction, protein localization, and membrane-bound versus water-soluble tasks. Besides, Rives *et al.* (2021) also reported better performance of Transformers compared to LSTM on contact prediction. But, to the best of our knowledge, no previous work tested Transformers on a functional annotation task that can be evaluated as accurately as enzyme functional annotation.

In addition to functional annotation, we also chose to evaluate the benefits of the Transformers’ attention mechanism as an interpretability method. In a word, for a given residue, attention provides an importance score showing which other residues are mostly related to it to perform a given prediction task. As such, attention naturally highlights key relations within the sequence (Chefer *et al.* 2021). Previous work (Vig *et al.* 2020) has related protein attention of Transformers [TAPE—Rao *et al.* (2019), ProtTrans—Elnaggar *et al.* (2022)] and the presence of binding sites. Yet, while these studies show the potential of attention-based methods, whether they can be used and be better than classical interpretability methods in the context of biological sequences is still an open question. Tackling this issue requires developing a common setup to allow for an exhaustive comparison between methods: this article presents such a setup in the context of enzymes’ functional annotation.

To summarize, our contribution is as follows: (i) we show that using Transformer neural networks achieves state-of-the-art results for predicting the enzymatic function of a protein from its sequence only; (ii) we present a simple attention-based interpretability method that outperforms classical generic ones in terms of coherence with prior biological knowledge.

## 2 Materials and methods

### 2.1 Enzyme class prediction task

#### 2.1.1 Task description and datasets

The task of interest in this article consists in predicting EC numbers at a specific level. This nomenclature is composed of four levels. The first level provides the main class of the enzyme, which is encoded by a number between 1 and 7. The second and third digits correspond respectively to the subclass and sub-subclass of the enzyme, and the last one represents specific metabolites and co-factors involved, which basically provides the actual reaction catalyzed or a restricted set of very similar reactions.

Three datasets, EC40, ECPred40, and DeepEC40, were studied in this article, in order to have the most direct comparison with state-of-the-art models. The first dataset, EC40 from Strodthoff *et al.* (2020), goes up to EC level two. It ensures that (i) sequences from the testing set share less than 40% sequence identity with any sequence used for training the models and (ii) sequences in the testing set share less than 40% sequence identity between themselves. We built ourselves the ECPred40 and DeepEC40 datasets, which include up to EC level four, for the comparison with ECPred (Dalkiran *et al.* 2018) and DeepEC (Ryu *et al.* 2019), respectively. We followed the time-based evaluation from Dalkiran *et al.* (2018) and made sure that test sequences share less than 40% sequence identity with training sequences as in Strodthoff *et al.* (2020). More specifically, we first retrieved the newly created annotations between Swiss-Prot releases used to train the models (release 2017_3 for ECPred40 and release 2018_1 for DeepEC40 in the original papers) and the latest release of Swiss-Prot (release: 2021_04). To ensure the identity threshold, we clustered these novel annotations, together with the training set, using MMseqs2 (Steinegger and Söding 2017) at 40% identity threshold: if two sequences shared 40% or more identity, they were assigned to the same cluster. To build the testing set, all clusters with at least one sequence from the training set were discarded and a representative of each remaining cluster was included in the testing set, ensuring less than 40% sequence identities between the sequences from the testing set and the other sequences used (training/validation). Concerning the training and validation sets, Dalkiran *et al.* (2018) applied the same process, keeping only one representative sequence per cluster. In contrast, we choose to keep all the sequences for training, as did Strodthoff *et al.* (2020).

We also labeled as ‘non-enzymes’ all sequences having neither GO annotation with catalytic activities, nor any EC number annotation. This is required to perform the prediction of whether a protein is an enzyme or not, referred to hereafter as ‘level 0’ prediction. For a fair comparison, we only kept the EC classes that the different models were capable of predicting (634 and 4669 classes at level 4 respectively for ECPred and DeepEC). We also filtered each dataset to fit the requirements in terms of sequence length, for ECPred (more than 40 AA, up to 1024 AA for memory constraints on our side) and DeepEC (between 40 and 1000 AA). We named these custom datasets ECPred40 and DeepEC40. Table 1 summarizes the number of protein sequences by classes in the different datasets. One can notice that the translocase class is only present in the SwissProt_2021_04 dataset since this class was only introduced in August 2018.

**Table 1.** Number of protein sequences in each EC first-level class for each dataset.^a^

Dataset	Not an enzyme	Oxidoreductases	Transferases	Hydrolases	Lyases	Isomerases	Ligases	Translocase	Total
EC40 training	0	14 574	34 031	25 329	8792	5399	11 861	0	99 986
EC40 validation	0	365	873	746	182	115	225	0	2506
EC40 testing	0	376	887	677	147	156	263	0	2506
ECPred40 training	22 797	21 380	62 124	33 362	16 926	10 926	24 045	0	191 560
ECPred40 validation	5502	3014	8097	3947	2008	1366	2536	0	26 470
ECPred40 testing	499	17	201	162	19	20	6	0	924
DeepEC40 training	24 8320	26 429	64 528	37 472	18 043	10 931	23 182	0	428 905
DeepEC40 testing	2164	48	184	182	21	13	6	0	2618
SwissProt_2021_04	302 753	26 278	80 485	40 913	23 253	14 453	26 374	8455	522 964

a The EC40 dataset is taken from Strodthoff *et al.* (2020). For the ECPred40 and the DeepEC40 datasets, training sets are from the original papers (Dalkiran *et al.* 2018, Ryu *et al.* 2019). Testing sets are built for a time-based evaluation, considering newly added sequences in SwissProt_2021_04. SwissProt_2021_04 is directly extracted from UniProtKB/SwissProt without filtering.

#### 2.1.2 Evaluation procedure and metrics for prediction performance

For the comparison with UDSMProt (Strodthoff *et al.* 2020), we used their EC40 dataset and computed the accuracy at level 2 as they did. We also computed the macro-F1 score for our model to have a basis for comparison that is not biased toward more common classes. Macro means that the average is computed on each enzyme class of the different metrics, which allows sampling biases to be mitigated if some enzyme classes are more represented than others. For the comparison with ECPred (Dalkiran *et al.* 2018), we also needed to evaluate the enzyme/non-enzyme discrimination. We have done two evaluations based on the new EC40Pred testing set. The first one focused on the enzyme versus non-enzyme classification task. For the second, we only considered the enzymes in the testing set and evaluated the predictions at levels 1 to 4. For DeepEC (Ryu *et al.* 2019), predictions directly span from level 0 (enzyme versus non-enzyme) to level 4. Macro measures (precision, recall, *F*1) were used.

Note that we also designed a dataset to compare our model EnzBert with DEEPre from Li *et al.* (2018). However, the server providing predictions for DeepPre was not functional at the time of our study. Hence, we could not include the corresponding benchmark.

Finally, we also compared our model to BLASTp (Altschul *et al.* 1990), to assess how alignment-based methods could compete. BLASTp was evaluated on the DeepEC40 dataset to make use of the most recent SwissProt release, thus providing more annotations for training.

### 2.2 The model

#### 2.2.1 The Transformer architecture

For better readability, we will use the term ‘Transformer’ instead of ‘Transformer Encoder’, as in Devlin *et al.* (2019). As input, Transformers usually take a sequence of ‘tokens’ of interest. Here, the tokens will be the various amino acids from a sequence as well as some special tokens used for training, as in Elnaggar *et al.* (2022). For each of these symbolic tokens, our Transformer will then learn an appropriate embedding, i.e. a continuous vector representing each of them.

Finally, amino acid sequence order is important but not taken into account by default (since Transformers are position invariant). Thus, it is needed to add positional vectors to each embedding at each position. In our model, positional encoding is a fixed sinusoidal vector (see Devlin *et al.* 2019).

**Figure 1.**
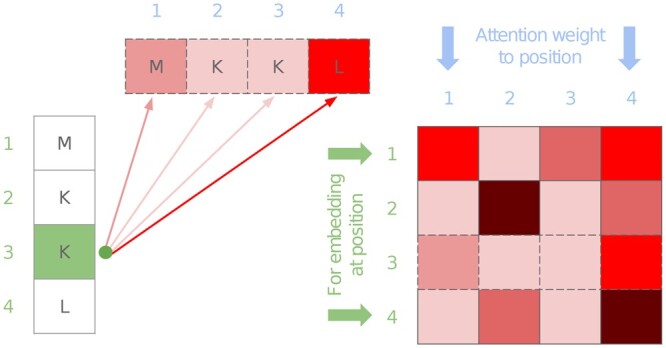
Given a sequence of four residues, the lysine (K) residue at position 3 gives the highest attention to the leucine (L) residue at position 4 (left). This attention row can be found on line 3 of the attention map on the right

One of the key aspects of the Transformer is the attention mechanism. As shown in Fig. 1, an attention head can be represented as a matrix. Row *i* in this matrix represents the weights given to other tokens to produce an embedding of token *i*. Each layer in the architecture presents multiple attention heads and the resulting embedding is passed on to the next layer.

#### 2.2.2 EnzBert

Our EnzBert models are based on a Transformer architecture. To avoid some unnecessary computation, we started from an already pre-trained Transformer, the ProtBert-BFD model variant from Elnaggar *et al.* (2022).

Then, we fine-tuned these models for the EC class prediction task on the different training sets to obtain the different EnzBert models. In order to do this, we classically appended a special (CLS) token to all input token sequences. The embedding of the (CLS) token is meant to represent the whole sequence, from which we can derive the functional annotation [as in Devlin *et al.* (2019)]. We did not freeze the first layers when performing the fine-tuning part, in contrast with Elnaggar *et al.* (2022). We used a normalization layer to speed up the training process, followed by a linear layer to project the (CLS) final embedding to the desired number of classes. For fine-tuning, we did not consider the EC hierarchy: all classes were treated as being independent, allowing us to use a cross-entropy loss function.

The code used for this article is written in PyTorch and is available at https://gitlab.inria.fr/nbuton/tfpc, the hyper-parameters are shown in Table 2.

**Table 2.** Hyper-parameters used for the fine-tuning of the three different versions of EnzBert.^a^

Parameter	EnzBert_EC40-ECPred40-SwissProt_
Dropout on (CLS)	0.2
Batch size	2
Accumulation step	16
Learning rate	1.0 × 10^−5^
Optimizer	Adam(β_1_ = 0.9, β_2_ = 0.999
Lr scheduler	Lr (epoch) = 0.8 × Lr (epoch − 1)
Number epochs	5-15-15

a When different a ‘-’ is used.

Three models were trained: EnzBert${}_{\text{EC}40}$, EnzBert${}_{\text{ECPred}40}$, and EnzBert${}_{\text{DeepEC}40}$ fine-tuned respectively on EC40, ECPred40, and DeepEC40 training sets. We also trained EnzBert${}_{\text{SwissProt}}$ on all SwissProt (release dump 2021_04) in order to compare interpretability methods.

Due to the presence of the over-represented ‘non-enzyme’ class (more examples of ‘non-enzymes’ than examples of other levels 4 EC) and EC classes with very few examples, we chose to balance the dataset during training with a weighted random sampler (WeightedRandomSampler class from PyTorch) to train EnzBert${}_{\text{SwissProt}}$. The chosen weights were the inverse of the occurrence for each class.

### 2.3 Interpretability

In order to compare our custom attention-based interpretability method, we first re-implemented classical methods on the same common setup for proper comparison.

#### 2.3.1 Descriptions of classical interpretability methods

**Figure 2.**
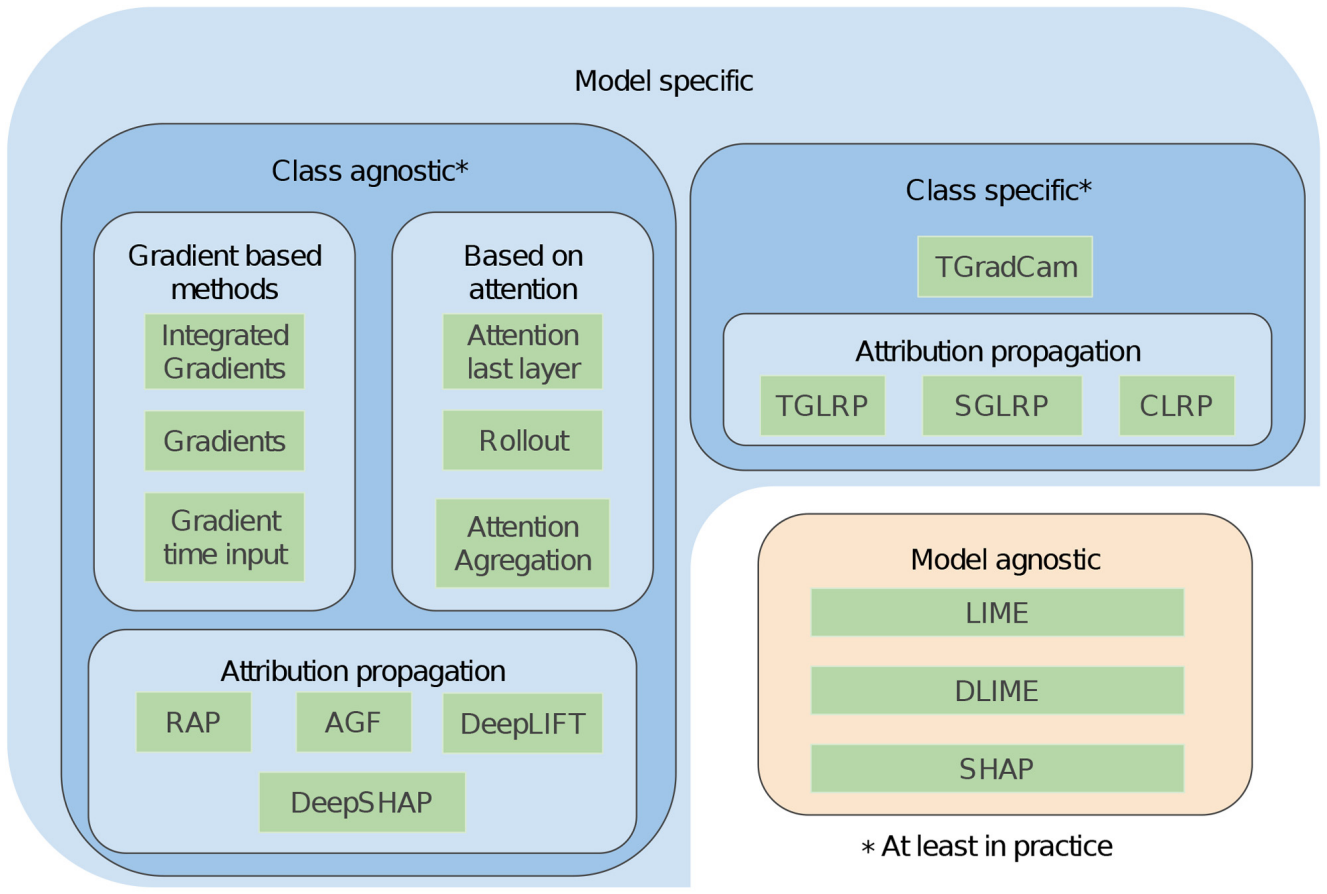
Summary of the main types of interpretability methods

As shown in Fig. 2, we considered methods that are model-specific (e.g. requiring the model to be differentiable) or model-agnostic, such as LIME (Ribeiro *et al.* 2016). We also included class-specific (e.g. TGradCam—see Chefer *et al.* 2021) and class-agnostic methods [e.g. Attention last layer, named Raw attention in Abnar and Zuidema (2020)]. Note that, while being class-specific in theory, generic Gradient-based methods tend to behave like class agnostic methods (Chefer *et al.* 2021), meaning that the feature importance does not change much between classes. Providing an exhaustive review of gradient-based interpretability methods is beyond the scope, as many variants exist [e.g. Gradient time input (Shrikumar *et al.* 2017), Integrated Gradient (Sundararajan *et al.* 2017), etc.].

Finally, some methods rely on the attention maps which are specific to Transformers (e.g. Attention Last Layer and Rollout, see Abnar and Zuidema 2020).

#### 2.3.2 Attention aggregation interpretability methods

All attention maps in the Transformer can be stored in a tensor of shape (L×H,N,N), with *L* the number of layers in the Transformer, *H* the number of heads per layer, and *N* the length of the sequences. The first dimension is the dimension of all heads for all layers, the second dimension is the attention received by a given residue from the others, and the last dimension is the attention given by a residue to all the others.

**Figure 3.**
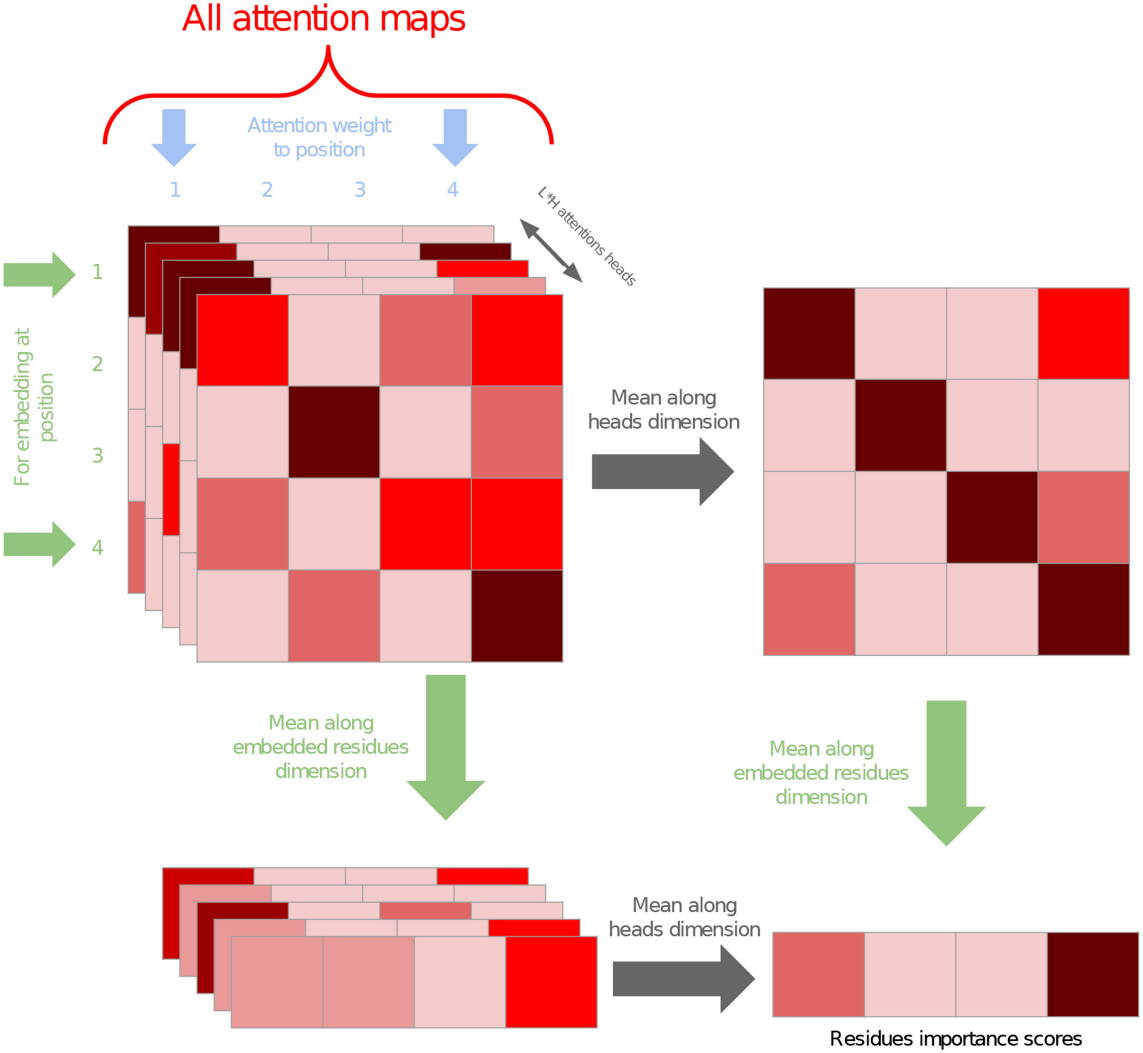
Aggregation methods AttnAgg1A1A and AttnAgg2A1A. AttnAgg1A1A averages over the first dimension (heads) and then averages over the first remaining dimension (attention over tokens) to obtain a row vector. AttnAgg2A1A averages first over the second (attention over tokens) and then averages over the first remaining dimensions (heads)

In order to obtain a vector of size *N* containing the ‘aggregated attention’ for each residue, we could collapse the T tensor along the second dimension [to obtain a matrix of shape (L×H,N)] and then along the first dimension of the resulting matrix, to obtain a vector of size *N* (as illustrated in Fig. 3), as well as other orders of aggregation, e.g. starting the aggregation on the first dimension of the tensor. In terms of aggregation, we explored two possibilities: the average and the maximum. Overall, combining along the various dimensions and choosing an aggregation method provides 13 different possibilities, after removing duplicates (16 before). We will name these variants AttnAgg followed by the dimension collapsed on the tensor and then the dimension collapsed on the resulting matrix, each annotated by the function used for pooling (A: average and M: max), e.g. AttnAgg1A1A as shown in Fig. 3.

#### 2.3.3 Catalytic residues as gold label

One key question is whether the residue importance for the classification task can be related to known features of the amino acids in the sequence. To explore this aspect, we used the mechanism and catalytic site atlas (M-CSA) (Ribeiro *et al.* 2018) database. It documents numerous known enzyme catalytic residues and reaction mechanisms. All information in this database has been manually curated and is supported by research papers. It describes 992 enzymes, with an average length of 439 amino acids and on average 4.9 catalytic residue annotations per enzyme.

These catalytic residues will be used as a proxy to evaluate the different interpretability methods and will be referred to as ‘gold labels’ in the following sections.

#### 2.3.4 Evaluation and metrics for interpretability

There is no clear consensus about which metric to use to evaluate interpretability with gold labels (DeYoung *et al.* 2020). In this article, we use mainly two types of curves, from which we derived comparison metrics. First, we took inspiration from Chefer *et al.* (2021). For a given interpretability method, for each enzyme, we selected the residues with the top-*k* importance scores given by the interpretability method. We then crossed this feature’s importance list with the gold labels: each important residue with a gold label is considered a true positive. This allowed us to compute an *F*1 score for a given sequence. We then averaged the *F*1 scores overall sequences and repeated this process from *k* = 1 to 50 top-*k* scores. Second, from the same importance list, we also derived a precision–recall curve. Such a curve is often used in the context of unbalanced classes, which is the case here as catalytic residues only represent 1.16% of all residues.

We then derived two metrics to quantitatively compare models. First, we considered the precision–recall gain area under the curve (PRG-AUC—Flach and Kull 2015). Second, we computed a maximum *F*-Gain score as follows. First, for each sequence, we deleted the score on the CLS token and rescaled the tokens’ importance score (either min-max scaling, normalization, division by L1 or L2 norms, or no scaling). Then, we created a set with all the (rescaled) scores of all the tokens from the testing set sequences. All tokens whose score is higher than a given threshold are considered ‘important’. An *F*1 score is then computed, crossing important tokens and known catalytic annotations. The maximum *F*1 score consists in keeping the highest *F*1 score achieved by varying the threshold. Finally, the *F*-Gain, precision gain and recall gain scores are derived from these *F*1, precision and recall scores accounting for the performances of an always-positive classifier (Flach and Kull 2015).

We also designed a ‘baseline’ method to check whether the distribution of scores was enough to provide high interpretability metrics. To do so, we shuffled the importance score of the tokens and applied the same metrics (PRG-AUC and maximum *F*-Gain score) to compare them to our (non-shuffled) results.

Computation time for each interpretability method was measured on a CPU as some interpretability methods (e.g. TGLRP) need more RAM than available in the GPUs at our disposal. All the models and the interpretability annotation are available on GitLab https://gitlab.inria.fr/nbuton/tfpc.

## 3 Results

### 3.1 Enzyme class prediction

Table 3 summarizes the class prediction quality of EnzBert_EC40_ on the EC40 test set, as well as UDSMProt (Strodthoff *et al.* 2020), the best-known enzyme predictor at level 2 using only sequences. On this dataset with less than 40% identity between training and testing sequences, the results show (i) an increase in accuracy from 87% for the previous state-of-the-art model, UDSMProt, to 97% for EnzBert_EC40_ concerning the prediction of the six possible classes at level 1 and (ii) an increase from 84% to 95% accuracy for the prediction of the 51 possible classes at level 2. Macro-*F*1, which allows all classes to be considered equally and avoids skewing the evaluation towards the most common classes, reaches here respectively 96% and 89% at levels 1 and 2 for EnzBert_EC40_.

**Table 3.** Comparison with UDSMProt of the prediction quality at the two levels of EC40 test set.

Model	Level	Macro-*F*1	Macro-precision	Macro-recall	Accuracy	Number of classes
UDSMProt	1				0.87	6
**EnzBert_EC40_**	1	0.96	0.96	0.96	**0.97**	6
UDSMProt	2				0.84	65
**EnzBert_EC40_**	2	0.89	0.93	0.87	**0.95**	65

When it comes to finer levels of predictions, the LSTM-based network from UDSMProt cannot be used as it is trained to predict up to level 2 classes only. The current potential state-of-the-art models for level 4 predictions are ECPred (Dalkiran *et al.* 2018) and DeepEC (Ryu *et al.* 2019) (they have not been formally compared to each other). Table 4 shows the prediction performances of EnzBert_ECPred40_ and ECPred at the different EC levels, from level 0 (enzyme vs non-enzyme discrimination task) to level 4 (634 classes), on ECPred40 test set. EnzBert_ECPred40_ improves predictions over ECPred at each level, except at level 1 where ECPred favors recall and our model favors precision. At level 4, the finer and more challenging level of prediction, our model improves both macro-precision and macro-recall with respect to ECPred, resulting in an increase of the macro-*F*1 score from 40.7% to 55.2%.

**Table 4.** Comparison with ECPred of the prediction quality at the five levels of ECPred40 test set.

Model	Level	Macro-F1	Macro-precision	Macro-recall	Accuracy	Number of classes
ECPred	0	0.769	0.784	0.781	0.769	2
**EnzBert_ECPred40_**	0	**0.837**	**0.874**	**0.831**	**0.845**	2
**ECPred**	1	**0.728**	0.691	**0.841**	**0.824**	6
EnzBert*_ECPred40_*	1	0.604	**0.784**	0.582	0.813	6
ECPred	2	0.492	0.468	0.579	0.759	51
**EnzBert_ECPred40_**	2	**0.629**	**0.676**	**0.672**	**0.781**	51
ECPred	3	0.496	0.491	0.549	0.727	132
**EnzBert_ECPred40_**	3	**0.609**	**0.625**	**0.652**	**0.749**	132
ECPred	4	0.407	0.431	0.412	0.636	634
**EnzBert_ECPred40_**	4	**0.552**	**0.576**	**0.562**	**0.687**	634

EnzBert_DeepEC40_ demonstrated better performance across the different EC levels compared to DeepEC. At level 4, DeepEC achieved a macro-*F*1 score of 20.5%, while EnzBert_DeepEC40_ achieved a score of 26.4%. For other results, please refer to the top of Table 5.

**Table 5.** Comparison with the macro-*F*1 between EnzBert${}_{\text{DeepEC}40}$, DeepEC, and BLASTp of the prediction quality at the five levels without enzyme *a priori* on the DeepEC40 dataset.

Model name	Level 0	Level 1	Level 2	Level 3	Level 4
DeepEC	0.662	0.408	0.378	0.324	0.205
BLASTp	0.697	0.439	0.408	0.354	0.214
**EnzBert** ${}_{\mathrm{DeepEC40}}$	**0.812**	**0.654**	**0.542**	**0.505**	**0.264**
**Number of classes**	2	8	53	131	547

In our approach, we focused on state-of-the-art models. Other tools exist like EzyPred (Shen and Chou 2007), EFICAz (Kumar and Skolnick 2012), but experimentation by Dalkiran *et al.* (2018) suggests that ECPred outperforms them. Moreover, Strodthoff *et al.* (2020) showed that UDSMprot is on par with ECPred at level 1 [level 2 performance of ECPred was not mentioned in Strodthoff *et al.*’s (2020) study]. These results suggest that Transformers outperform state-of-the-art tools for the prediction of the enzymatic class of proteins from their sequence. In the next section, we focus on the interpretability offered by their attention mechanism.

### 3.2 Interpretability

**Figure 4.**
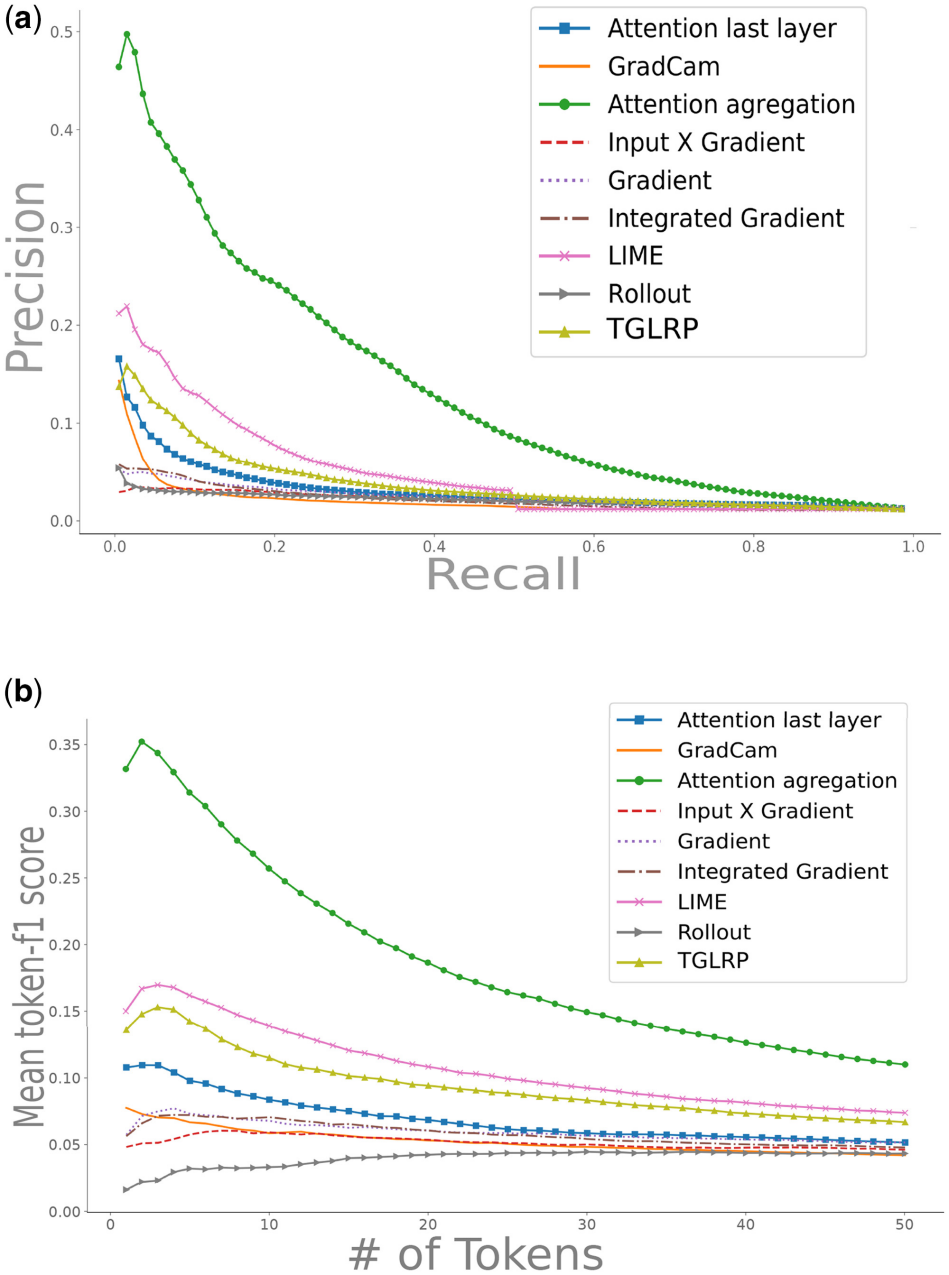
(a) Precision/recall curve for different interpretability methods. The attention aggregation group is represented by the AttnAgg1A1A method. (b) *F*1 score for top-*k* residue with the highest residue importance scores

The Transformers’ attention provides a built-in interpretation mechanism, but its complexity in the case of multiple attention heads makes it difficult to use directly. We have thus proposed and tested different attention aggregation methods. Among 13 different possibilities for attention aggregation described in Section 2.3.2, the best performing was ‘AttnAgg1A1A’, described in Fig. 3. It consists in (i) taking the average over all attention maps and then (ii) averaging over the vertical dimension of this average attention map. This aggregation method can be shown to be equivalent to AttnAgg2A1A, as it applies the average operator twice and is always performed on the same number of elements. Exploring other aggregation orders shows that averaging over the attention a given residue is paying to all others leads to poor performance (all PRG-AUC < 90 %). In contrast, all methods focusing on the attention received by a given residue perform well (PRG-AUC > 90 %), with the exception of AttnAgg1M1M (PRG-AUC 66.25%). Note that this method chains two max pooling operations instead of averaging, which might result in a loss of information. As shown in Fig. 4a, the best attention aggregation method outperforms all other interpretability methods, for all levels of recall. In the low recall regime, AttnAgg1A1A shows at least twice the precision of all other methods.

Figure 4a shows the *F*1 score with respect to the *k* most important residues identified by each interpretability method (see Section 2.3). AttnAgg1A1A outperforms all other interpretability methods for all numbers of residues between 1 and 50. As an illustration, the highest point in Fig. 4b, for Attention aggregation corresponds to two residues: this means that testing whether the two most important residues are catalytic sites results in an *F*1 score of 35%. The same procedure with the two most important residues identified by LIME, for instance, results in an *F*1 score of 19%. Finally, in Table 6, we reported the PRG-AUC, the max *F*-Gain metrics for each method. AttnAgg1A1A outperforms the other methods on both prediction metrics.

**Table 6.** Evaluation of best interpretability method of each category with respect to the M-CSA dataset.^a^

Method type	PRG-AUC (×100)	Max *F*-Gain (%)	Time (s)
Random	42.54±4.37	69.85±1.04	
Grad	75.01	81.27	4.64
Grad X input	63.62	78.66	7.74
Integrated grad	76.41	81.70	$2.48\times 10^{2}$
Attn last layer	87.80	85.62	**2.87**
**Attn agg**	**98.02**	**96.05**	3.72
Rollout	66.08	76.77	2.95
TGLRP	90.92	88.56	$4.05\times 10^{1}$
TGradCam	81.00	76.77	$4.35\times 10^{1}$
LIME	93.46	91.44	$1.73\times 10^{4}$

a PRG-AUC and the max *F*-Gain metrics are reported, and we also report the mean execution time for each method [for one protein, in second on Intel(R) Core(TM) i7-10610U CPU @ 1.80 GHz].

Considering the execution time, three groups appear and AttnAgg1A1A belongs to the fastest one. The first group contains methods nearly as quick as classical prediction, all being under 5 s to run: Gradient, Gradient time input, attention last layer and attention aggregation. A second group consists of methods around 10 times slower, with TGLRP, GradCam, and integrated gradient. Finally, the LIME method is about 5000 times slower than prediction times, which corresponds to the number of sequences needed to estimate the local estimator of LIME. For instance, evaluating the importance of residues in 1000 proteins of length 439, the average length in the M-CSA database would take about 197 days with LIME and less than an hour with our approach on CPU.

#### 3.2.1 Visualization of important residues

**Figure 5.**
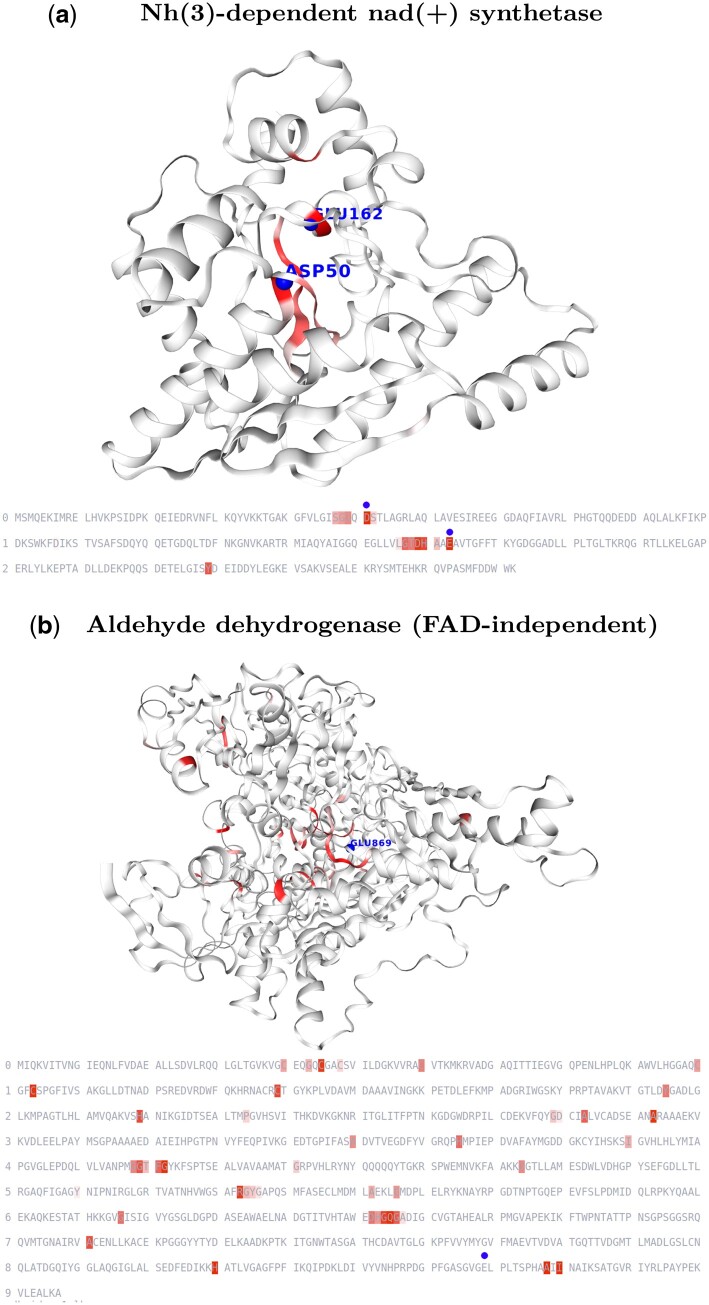
3D and 1D positions of most important residues highlighted by our interpretability method AttnAgg1A1A on NH(3)-dependent NAD(+) synthetase, one of the best examples of catalytic site retrieval, and aldehyde dehydrogenase (FAD-independent), the worst example of catalytic site retrieval. The 5% most important residues for our interpretability method are highlighted and catalytic sites identified in the M-CSA database are represented by spheres

Figure 5 illustrates cases of best and worst agreement between the known annotation for a given residue to be a catalytic site and the corresponding importance score for the same residue computed with our best interpretability method, namely AttnAgg1A1A.

In the best case, seven proteins exhibit a PRG-AUC of one, which means that all their catalytic residues have the highest importance scores. Among those, we chose to present the NH(3)-dependent NAD(+) synthetase enzyme (Uniprot AC P08164, M-CSA ID 200) in Fig. 5 since it has the highest number of catalytic sites (two) and the highest annotation quality of 5/5 in UniProt. The two highest importance scores correspond to the two catalytic residues: GLU162 and ASP50. The two next highest importance scores correspond to two residues ASP158, and HIS159 which are near the first catalytic site. We then searched in Swiss-Prot (the manually reviewed part of UniProtKB) for potential annotations in the other residues within the top 10 importance scores. We found that four were associated with binding sites: GLY48, GLY47, SER46, and THR157. Overall, catalytic site and binding site annotations were significantly enriched in the top 10 highest importance scores (6/10 compared to 21/271 for the whole enzyme, Chi-squared test, *P* value of .00003).

The worst agreement between importance score and the presence of catalytic sites was found for the aldehyde dehydrogenase (FAD-independent) enzyme (Uniprot AC Q46509, M-CSA ID 105). The sole catalytic residue, GLU869, is not highlighted by our interpretability method, that is, it does not belong to the top 5% residues in terms of importance scores (position 887 over 907 residues). In this case, residue importance scores seem to focus more on the binding sites of the protein. Indeed, two binding sites documented in SwissProt are found in the top 10 residues in terms of importance score: CYS103 and CYS45. In this case as well, we observe a significant enrichment of binding and catalytic sites within the top 10 highest importance scores (2/10 compared to 10/907 for the whole enzyme, Chi-squared test, *P* value of .00516).

## 4 Discussion

Our experimentation shows that the use of attention has the potential to outperform state-of-the-art approaches for the prediction of the enzymatic class based on sequences. This provides another example, outside the field of NLP, of the interest of Transformers over LSTM-based neural networks. This study also demonstrates the difficulty of estimating the performances of models. Indeed, although we took special care to evaluate the approaches on published and functional benchmarks—the EC40 benchmark from Strodthoff *et al.* (2020) and the new benchmark ECPred40 up to level 4 inspired by the time-based evaluation by Dalkiran *et al.* (2018)—a considerable difference in the expected performances can be observed at levels 1 and 2 with respect to the dataset used, despite the fact that they are based on the same identity threshold of 40% (see Tables 3 and 4). As an additional experiment (data not shown), we trained an EnzBert model to predict the level 2 classes (without the non-enzyme class) on ECPred40 with exactly the same procedure as for EC40. The gap in classification performance was still significant. This highlights how critical the choice of the dataset is and that further studies will probably be needed to better estimate the actual predictive power of enzyme classification methods in practice.

Our experimentation also shows that the attention of Transformers provides a built-in interpretable mechanism pointing to important residues of enzymes, thanks to our simple AttnAgg1A1A aggregation of multi-head attentions. It is surprising that this simple linear aggregation retrieves enzymatic sites better than state-of-the-art attention-based interpretability methods. For instance, the best of these later methods, which has also been efficient in text interpretability (Chefer *et al.* 2021), is TGLRP. Based on attention maps and gradient computations, TGLRP can take into account non-linearities and might focus on more subtle signals. The characterization of its results and their comparison to the important non-catalytic sites found by our method remains an open issue. More generally, the study focused here on the safest, but limited, proxy for estimating the value of the different interpretability methods and it would be interesting to study other important residue features, as suggested by the interpretation of Fig. 5.

## 5 Conclusion and perspective

We provide a state-of-the-art model *EnzBert* that only uses sequences to predict enzymes’ functional annotation. This model benefits from the attention mechanism through the use of the Transformer architecture. We also propose a simple yet successful interpretability method that only relies on attention maps. The resulting insights on enzyme sequences can help further research to better understand how enzyme classes are derived from the protein sequence and even help for further steps, for example regarding enzyme optimization.

Finally, in our model, enzymatic classes are considered independent from one another. This means that we do not yet exploit to the fullest the underlying hierarchy structure of EC numbers. Integrating meaningful prior knowledge into deep neural network architectures remains challenging but we believe that the field of automatic functional annotation might particularly benefit from such approaches.

## Data Availability

Source code and datasets are respectively available at https://gitlab.inria.fr/nbuton/tfpc and https://doi.org/10.5281/zenodo.7253910

## References

[btad620-B1] Abnar S , Zuidema W. Quantifying Attention Flow in Transformers. In: *Proceedings of the 58th Annual Meeting of the Association for Computational Linguistics*, 4190–7. Online: Association for Computational Linguistics, 2020.

[btad620-B2] Altschul SF , Gish W, Miller W et al Basic local alignment search tool. J Mol Biol 1990;215:403–10. 2231712 10.1016/S0022-2836(05)80360-2

[btad620-B3] Baldazzi D , Savojardo C, Martelli PL et al BENZ WS: the Bologna ENZyme Web Server for four-level EC number annotation. Nucleic Acids Res 2021;49:W60–6. 33963861 10.1093/nar/gkab328 PMC8262719

[btad620-B4] Borro LC , Oliveira SRM, Yamagishi MEB et al Predicting enzyme class from protein structure using Bayesian classification. Genet Mol Res 2006;5:193–202. 16755510

[btad620-B5] Chefer H , Gur S, Wolf L. Transformer Interpretability Beyond Attention Visualization. In: *IEEE/CVF Conference on Computer Vision and Pattern Recognition (CVPR), Nashville, TN, USA, 2021*, 782–91. 2021.

[btad620-B6] Dalkiran A , Rifaioglu AS, Martin MJ et al ECPred: a tool for the prediction of the enzymatic functions of protein sequences based on the EC nomenclature. BMC Bioinformatics 2018;19:334. 30241466 10.1186/s12859-018-2368-y PMC6150975

[btad620-B7] De Ferrari L , Aitken S, van Hemert J et al EnzML: multi-label prediction of enzyme classes using InterPro signatures. BMC Bioinformatics 2012;13:61. 22533924 10.1186/1471-2105-13-61 PMC3483700

[btad620-B8] Devlin J , Chang M-W, Lee K et al *BERT: Pre-training of Deep Bidirectional Transformers for Language Understanding*. North American Chapter of the Association for Computational Linguistics,2019.

[btad620-B9] DeYoung J , Jain S, Rajani NF et al ERASER: A Benchmark to Evaluate Rationalized NLP Models. In: *Proceedings of the 58th Annual Meeting of the Association for Computational Linguistics*, 4443–58. Online: Association for Computational Linguistics, 2020.

[btad620-B10] Elnaggar A , Heinzinger M, Dallago C et al ProtTrans: toward understanding the language of life through self-supervised learning. IEEE Trans Pattern Anal Mach Intell 2022;44:7112–27. 34232869 10.1109/TPAMI.2021.3095381

[btad620-B11] Flach PA , Kull M. Precision-recall-gain curves: PR analysis done right. In: *Proceedings of the 28th International Conference on Neural Information Processing Systems—Volume 1, NIPS’15*, 838–46. Cambridge, MA: MIT Press, 2015.

[btad620-B12] Kumar N , Skolnick J. EFICAz2.5: application of a high-precision enzyme function predictor to 396 proteomes. Bioinformatics 2012;28:2687–8. 22923291 10.1093/bioinformatics/bts510 PMC3467752

[btad620-B13] Li Y , Wang S, Umarov R et al DEEPre: sequence-based enzyme EC number prediction by deep learning. Bioinformatics 2018;34:760–9. 29069344 10.1093/bioinformatics/btx680 PMC6030869

[btad620-B14] Matsuta Y , Ito M, Tohsato Y. ECOH: an Enzyme Commission number predictor using mutual information and a support vector machine. Bioinformatics 2013;29:365–72. 23220570 10.1093/bioinformatics/bts700

[btad620-B15] Merity S , Keskar NS, Socher R. Regularizing and Optimizing LSTM Language Models. In: *International Conference on Learning Representations*, 2018.

[btad620-B16] Nagao C , Nagano N, Mizuguchi K. Prediction of detailed enzyme functions and identification of specificity determining residues by random forests. PLoS One 2014;9:e84623. 24416252 10.1371/journal.pone.0084623 PMC3885575

[btad620-B17] Nallapareddy MV , Dwivedula R. ABLE: attention based learning for enzyme classification. Comput Biol Chem 2021;94:107558. 34481129 10.1016/j.compbiolchem.2021.107558

[btad620-B18] Qiu J-D , Luo S-H, Huang J-H et al Using support vector machines to distinguish enzymes: approached by incorporating wavelet transform. J Theor Biol 2009;256:625–31. 19049810 10.1016/j.jtbi.2008.10.026

[btad620-B19] Raffel C , Shazeer N, Roberts A et al Exploring the limits of transfer learning with a unified text-to-text transformer. *J Mach Learn Res* 2020;**21**:140.

[btad620-B20] Rao R , Bhattacharya N, Thomas N et al Evaluating Protein Transfer Learning with TAPE. *Adv Neural Inf Process Syst* 2019;**32**:9689–701. PMC7774645 33390682

[btad620-B21] Ribeiro A , Holliday GL, Furnham N et al Mechanism and catalytic site atlas (M-CSA): a database of enzyme reaction mechanisms and active sites. Nucleic Acids Res 2018;46:D618–23. 29106569 10.1093/nar/gkx1012 PMC5753290

[btad620-B22] Ribeiro MT , Singh S, Guestrin C. "Why Should I Trust You?": Explaining the Predictions of Any Classifier. In: *Proceedings of the 22nd ACM SIGKDD International Conference on Knowledge Discovery and Data Mining (KDD '16)*, 1135–44. New York, NY: Association for Computing Machinery, 2016.

[btad620-B23] Rives A , Meier J, Sercu T et al Biological structure and function emerge from scaling unsupervised learning to 250 million protein sequences. Proc Natl Acad Sci USA 2021;118:e2016239118. 33876751 10.1073/pnas.2016239118 PMC8053943

[btad620-B24] Ryu JY , Kim HU, Lee SY. Deep learning enables high-quality and high-throughput prediction of Enzyme Commission numbers. Proc Natl Acad Sci USA 2019;116:13996–4001. 31221760 10.1073/pnas.1821905116 PMC6628820

[btad620-B25] Sanderson T , Bileschi ML, Belanger D et al ProteInfer, deep neural networks for protein functional inference. Elife 2023;12:e80942. 36847334 10.7554/eLife.80942 PMC10063232

[btad620-B26] Schnoes AM , Ream DC, Thorman AW et al Biases in the experimental annotations of protein function and their effect on our understanding of protein function space. PLoS Comput Biol 2013;9:e1003063. 23737737 10.1371/journal.pcbi.1003063 PMC3667760

[btad620-B27] Shen H-B , Chou K-C. EzyPred: a top-down approach for predicting enzyme functional classes and subclasses. Biochem Biophys Res Commun 2007;364:53–9. 17931599 10.1016/j.bbrc.2007.09.098

[btad620-B28] Shrikumar A , Greenside P, Shcherbina A et al Learning important features through propagating activation differences. In: *Proceedings of the 34th International Conference on Machine Learning - Volume 70 (ICML'17)*, JMLR.org, 3145–53, 2017.

[btad620-B29] Steinegger M , Söding J. MMseqs2 enables sensitive protein sequence searching for the analysis of massive data sets. Nat Biotechnol 2017;35:1026–8. 29035372 10.1038/nbt.3988

[btad620-B30] Strodthoff N , Wagner P, Wenzel M et al UDSMProt: universal deep sequence models for protein classification. Bioinformatics 2020;36:2401–9. 31913448 10.1093/bioinformatics/btaa003 PMC7178389

[btad620-B31] Sundararajan M , Taly A, Yan Q. Axiomatic Attribution for Deep Networks. In: *International Conference on Machine Learning*, 2017.

[btad620-B32] The UniProt Consortium. UniProt: the universal protein knowledgebase in 2021. Nucleic Acids Research 2021;49:D480–9. 33237286 10.1093/nar/gkaa1100 PMC7778908

[btad620-B33] Vaswani A , Shazeer N, Parmar N et al Attention is all you need. In: *Proceedings of the 31st International Conference on Neural Information Processing Systems (NIPS'17)*, 6000–10. Red Hook, NY,: Curran Associates Inc., 2017.

[btad620-B34] Vig J , Madani A, Varshney LR et al \BERT\ology Meets Biology: Interpreting Attention in Protein Language Models. In: *International Conference on Learning Representations*, 2021

[btad620-B35] Volpato V , Adelfio A, Pollastri G. Accurate prediction of protein enzymatic class by N-to-1 neural networks. BMC Bioinformatics 2013;14 Suppl 1:S11. 10.1186/1471-2105-14-S1-S11 PMC3548677 23368876

[btad620-B36] Webb E. Enzyme nomenclature 1992. Recommendations of the Nomenclature Committee of the International Union of Biochemistry and Molecular Biology on the Nomenclature and Classification of Enzymes, 1992.

[btad620-B37] Yu T , Cui H, Li JC et al Enzyme function prediction using contrastive learning. Science 2023;379:1358–63. 36996195 10.1126/science.adf2465

[btad620-B38] Zhou N , Jiang Y, Bergquist TR et al The CAFA challenge reports improved protein function prediction and new functional annotations for hundreds of genes through experimental screens. Genome Biol 2019;20:244. 31744546 10.1186/s13059-019-1835-8 PMC6864930

